# #MindinBody - feasibility of vigorous exercise (Bikram yoga versus high intensity interval training) to improve persistent pain in women with a history of trauma: a pilot randomized control trial

**DOI:** 10.1186/s12906-019-2642-1

**Published:** 2019-08-29

**Authors:** Alison Flehr, Christopher Barton, Jan Coles, Stephen J. Gibson, Gavin W. Lambert, Elisabeth A. Lambert, Arup K. Dhar, John B. Dixon

**Affiliations:** 1 Department of General Practice, School of Primary and Allied Health Care, Faculty of Medicine, Nursing and Health Sciences, Monash University, Melbourne, VIC Australia; 2 Caulfield Pain Management and Research Centre, Caulfield Hospital, Caulfield, Australia; 3 Iverson Health Innovation Research Institute and School of Health Sciences, Swinburne University of Technology, Hawthorn, VIC Australia; 4 Human Neurotransmitter Laboratory, Baker IDI Heart and Diabetes Institute, Melbourne, Australia; 5 Primary Care Research, School of Primary and Allied Health Care, Faculty of Medicine, Nursing and Health Sciences, Monash University, Melbourne, VIC Australia; 6 Alfred Psychiatry, Alfred Health, Melbourne, VIC Australia; 7 Faculty of Medicine, Nursing and Health Sciences, Monash University, Melbourne, VIC Australia; 8 Clinical Obesity Research Laboratories, Baker IDI Heart and Diabetes Institute, Melbourne, Victoria Australia

**Keywords:** Persistent pain, Autonomic regulation, Allostatic load, Psychobiology, Vigorous exercise, Bikram yoga, HIIT, Sensorimotor retraining exposure therapy

## Abstract

**Background:**

The neurobiology of persistent pain shares common underlying psychobiology with that of traumatic stress. Modern treatments for traumatic stress often involve bottom-up sensorimotor retraining/exposure therapies, where breath, movement, balance and mindfulness, are used to target underlying psychobiology. Vigorous exercise, in particular Bikram yoga, combines many of these sensorimotor/exposure therapeutic features. However, there is very little research investigating the feasibility and efficacy of such treatments for targeting the underlying psychobiology of persistent pain.

**Methods:**

This study was a randomized controlled trail (RCT) comparing the efficacy of Bikram yoga versus high intensity interval training (HIIT), for improving persistent pain in women aged 20 to 50 years. The participants were 1:1 randomized to attend their assigned intervention, 3 times per week, for 8 weeks. The primary outcome measure was the Brief Pain Inventory (BPI) and further pain related biopsychosocial secondary outcomes, including SF-36 Medical Outcomes and heart rate variability (HRV), were also explored. Data was collected pre (t0) and post (t1) intervention via an online questionnaire and physiological testing.

**Results:**

A total of 34 women were recruited from the community. Analyses using ANCOVA demonstrated no significant difference in BPI (severity plus interference) scores between the Bikram yoga (*n* = 17) and the HIIT (*n* = 15). Women in the Bikram yoga group demonstrated significantly improved SF-36 subscale physical functioning: [ANCOVA: F(1, 29) = 6.17, *p* = .019, partial eta-squared effect size (η_p_^2^) = .175 and mental health: F(1, 29) = 9.09, *p* = .005, η_p_^2^ = .239; and increased heart rate variability (SDNN): F(1, 29) = 5.12, *p* = .013, η_p_^2^ = .150, scores compared to the HIIT group. Across both groups, pain was shown to decrease, no injuries were experienced and retention rates were 94% for Bikram yoga and 75% for HIIT .

**Conclusions:**

Bikram yoga does not appear a superior exercise compared to HIIT for persistent pain. However, imporvements in quality of life measures and indicator of better health were seen in the Bikram yoga group. The outcomes of the present study suggest vigorous exercise interventions in persistent pain cohorts are feasible.

**Trial registration:**

Australian New Zealand Clinical Trials Registry (ACTRN12617001507370, 26/10/2017).

**Electronic supplementary material:**

The online version of this article (10.1186/s12906-019-2642-1) contains supplementary material, which is available to authorized users.

## Background

Chronic pain, or persistent pain, is a significant problem globally with substantial impacts to individuals, their support networks, and society. The International Association for the Study of Pain (IASP) defines chronic pain as “pain that persists beyond the normal tissue healing time, usually ≥3 months, in the absence of an obvious underlying biological cause” [1]. Yet there is also a great deal of evidence associating chronic pain with precipitating events beyond that of specific tissue damage, specifically in association with interpersonal trauma. A systematic review and meta-analysis examining chronic widespread pain, in association with a range of traumatic events found participants exposed to traumatic events were 3.35 times more likely to suffer from chronic widespread pain [2]. Another study (*N* = 1152) reported the adjusted relative-risk of chronic pain and chronic pelvic pain in women exposed to psychological inter-partner violence to be 1.91 and 1.62 respectively [3]. Further reviews have reported that individuals with a history of sexual abuse were 2.2 times more likely to be diagnosed with non-specific chronic pain, and 2.73 times more likely to be diagnosed with chronic pelvic pain [4] and that such effects appear to be additional to those of physical injury-related pain [5].

It is widely accepted within the current pain literature that chronic pain is closely associated with traumatic stress outcomes [6], particularly post-traumatic stress disorder (PTSD) [7–9]. PTSD is a complex ‘Trauma and Stressor Related Disorder’ [10]. Current complex trauma theory emphasizes a psychobiological mechanistic model, that is, intense traumatic experience dysregulates the functioning of the autonomic nervous system and increases allostatic load [11, 12]; where allostatic load refers to the added burden (cost, and wear and tear) to the autonomically dysregulated system working to restore homeostatisis [13]. Certainly there is considerable evidence of associations of a dysregulated stress response and traumatic life experience [14–16] and in particular early life stress [17, 18]. However, autonomic dysregulation has also been implicated in the development and maintenance of chronic pain, said to “set up a feedback loop between pain and stress reactivity” [19]. Which suggests that when the persistent pain patient is autonomically dysregulated, they have increased allostatic load (biopsychosocial burden), which in turn negatively impacts persistent pain levels [20]. Yet there is very little research investigating the efficacy of psychobiological mechanistic modelled therapies for chronic pain (hence forth referred to as persistent pain).

Current psychobiological complex trauma therapies emphasize a bottom-up, right brain focus [21]. Such approaches include body-oriented therapies [22] and sensorimotor psychotherapy [23], where emotional regulation and mind-body integration is targeted through focused breath, movement, posture, touch, balance, and mindfulness. Such components have also been recognised to be present in yoga, and as a consequence, trauma sensitive yoga treatments and programs have emerged [24–27]. There have also been a number of RCTs investigating the efficacy of yoga for pain, specifically with regard to safety [28] and pain improvements [29]. However, such research is very much in its infancy and is hindered by methodological limitations, in particular, the lack of consistent, comparable forms of exercise [30]. Regardless, a number of yoga for pain programs have been developed [31]. The current trauma sensitive yoga and yoga for pain programs emphasize a gentle approach to the practice in an effort to promote relaxation and movement confidence in a minimally confronting way.

An alternative approach to target autonomic dysregulation may be bottom-up sensorimotor retraining/exposure therapy vigorous activity. Although sensorimotor retraining [32], and exposure based treatments [33] have previously been suggested to improve persistent pain, previous strategies have emphasized top-down processing and utilized exteroceptive stimuli. In this context, vigorous exercise, through bottom-up processing, would expose the pain patient to a high level of physical discomfort (interoceptive stimuli: rapid heart rate and respiration, muscle tension, perspiration), in a safe supportive environment. Exposure to safe and controlled physical discomfort will allow the pain patient to practice how they emotionally respond to physical and emotional sensations. Such practice can train the pain patient to better self-regulate, reducing their biopsychosocial burden, and by association, reducing persistent pain levels.

The primary aim of this feasibility study was to provide preliminary evidence of efficacy and assess feasibility of two types of vigorous exercise (Bikram yoga and high intensity interval training [HIIT]) as bottom-up sensorimotor retraining/exposure therapies to improve persistent pain severity and interference in women with persistent pain and a history of trauma. It was hypothesized that Bikram yoga would be the superior exercise as it possesses more core features of, and the focused mindfulness of, other psychobiological trauma therapies, while the HIIT does not. Second, the study aimed to explore the variability of impacts of Bikram yoga compared with HIIT on a range of persistent pain associated biopsychosocial factors. The outcomes of the study can be used to determine power and sample size for a full scale trial of vigorous exercise for persistent pain; and also provides evidence of the feasibility of vigorous exercise interventions for women with persistent pain and history of trauma.

## Method

### Design

The present study was a parallel, open-label, randomised control trial that compared Bikram yoga (experimental group) to high intensity interval training (HIIT) (control group), using a pre (t0)/post (t1) intervention design. Prior to beginning the intervention, participants were randomized in a 1:1 ratio into (1) an 8-week, 3 sessions per week, studio-run Bikram Yoga program or (2) an 8-week, 3 sessions per week, gym-run HIIT program. The study’s primary outcome measure was combined pain severity and interference as measured by the Brief Pain Inventory (BPI).

### Participants

Women aged 20 to 50 (adult non-menopausal women), with a persistent pain condition and a self-reported history of trauma (physiological [an incident resulting in tissue damage] and/or psychological [an incident resulting in emotional damage]), were recruited from the community. Adult non-menopausal women were chosen for this project to avoid the potential confounding effects of hormonal changes associated with the menopausal transition [34, 35]. Participant eligibility criteria are presented in Table 1. Respondents were asked to rate their average pain severity over the last week from 0 to 10 as per the Brief Pain Inventory (BPI) [36]. To be included in the study, participants needed to provide a score of 5 or greater, this was to allow for a 2 point minimum clinically important difference change in pain [37], and an average standard deviation of 2.35, calculated from a range of previous pain studies utilising the BPI measure in allied health interventions [38–41]. Regarding the self-reported history of trauma: no time frame was set on the experience of past trauma events. Rather if the participants felt they were still impacted by the event in the past 12 months - as per the Life Stressor Checklist questionnaire [42], they were eligible to take part in the current trial. Participants’ were recruited from Oct 2016 through to July 2017 from a variety of sources including: flyers in relevant community-based organizations/groups (e.g. community centres, libraries, non-government organisations); free and paid advertisements in print and online media; social media posts; and; flyers in allied health, medical, and mental health clinic waiting rooms. Study recruitment information directed interested women to contact the researcher either by telephone or email for more information. Prior to recruitment, all participants were required to provide medical clearance from their General Practitioner stating approval to complete the interventions and undertake a diagnostic psychiatric interview.

**Table 1:** Participant eligibility criteria

Inclusion Criteria	Exclusion Criteria
Female	-Malignant pain (pain associated with disease, cancer for example)
Aged 20 to 50 years old	-Bone fracture
Persistent pain	-Dislocated joint
-present for longer than 12 months	-Current diagnosis of
-assessed as average pain level over the last week	-Diabetes
	-Heart disease
A self-assessed history or trauma	-Hypertension
Willingness to be randomized to either arm of the study	-Endocrine disorder
	-Organic brain syndrome
	-Other cognitive dysfunction
	-BMI < 18 or > 40
	-Peri or post-menopausal
	-Breast feeding
	-Insufficient understanding of English
	-Unable to provide medical clearance to participant in an exercise intervention
	-Lack of consent
	-Unwillingness to be randomized
	-‘Moderate’ or ‘high’ for suicidality from the MINI International Neuropsychiatric Interview
	-Other severe medical and/or psychiatric comorbidities that prevent safe and/or adequate participation, assessed by the investigators on a case-by-case basis

### Study interventions, intervention 1: Bikram yoga

**Fig. 1:**
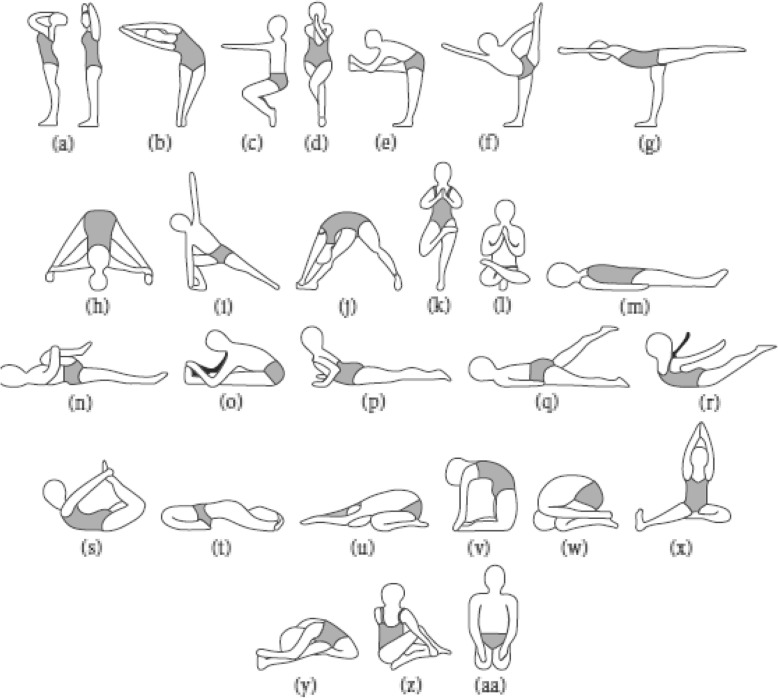
Bikram yoga sequence of asanas (poses). Standing pranayama (**a**), standing sequence (**b**–**l**), savasana (**m**), floor asanas (**n**–**z**) and final savasana (**aa**) [43]

Participants randomised to Bikram Yoga took part in an 8-week, 3 sessions per week, studio-run Bikram yoga program. All Bikram yoga classes used a set instructional dialogue that is strictly standardized across instructors and studio locations and were taught by certified Bikram yoga teachers (successful completion of Bikram yoga teacher training). Each Bikram yoga session consisted of the same series of 26 postures. “Every 90 min class begins with standing pranayama (deep breathing) followed by the standing sequence (45–50 min, Fig. 1a–l). The standing sequence is followed by a 2 min savasana (Fig. 1l and m) and a sequence of floor asanas (35–40 min, Fig. 1(n and aa). A 20-s savasana is taken between each asana in the floor series. Class finishes with a seated kapalabhati breathing exercise (i.e., quick, strong exhalations) and a final savasana.” [43]. Bikram yoga classes are undertaken in a room held at a constant heat of 40 degrees Celsius and humidity of 40% [43, 44]. Bikram yoga postures are classified as light-to-moderate by the American College of Sports Medicine [45], however, Bikram yoga intensities (although highly dependent on posture) have been reported to reach up to 6.0 metabolic equivalence of task [46], the threshold for vigorous activity intensity.

Although participants were encouraged to complete the entire 90 min series, it was emphasized to only do what was comfortable and to not to push themselves beyond their own physical limits, particularly in the initial sessions. They were also advised that the teachers were available to discuss modifications if anything triggered alarming discomfort. In addition, they were encouraged to be mindful of their physical exertion and to take breaks or sit postures out as required; this included their psychological limits as well [25]. Each participant was entered into the membership database of their studio of choice at check-in, before each class, so that attendance and compliance could be accurately tracked. Bikram class size average at 15 members per class but can range from 5 to 30.

### Intervention 2: HIIT

Participants randomised to HIIT took part in an 8-week, 3 sessions per week, gym-run HIIT program. HIIT classes were a trademarked Adrenaline HIT™ coach-team workout program focusing on functional movements within a small group (up to 15) setting [47]. Adrenaline HIT™ is a functional training that incorporates movement patterns that lend themselves to everyday activities including: running, throwing, standing from a seated position, placing things overhead and picking things up. Sessions are 45 min long, broken down into three 15 min sections: ‘Teach It’ - Warm up and Demonstration, ‘Do It’ - Complete the exercise with perfect technique, and ‘Beat It’ - Complete the workout at high intensity without compromising form. Adrenaline HIT™ consists of 4 workout formats; ‘Time’ – Complete the circuit as fast as possible, ‘Tabata’ – Interval training utilising 8 exercises followed by a cardio burst, ‘Reps’ – Do a maximum reps or load in a set time, ‘Box’ – 12 min to complete as many rounds of the 4 cross training stations followed by a cardio burst. [47]

The program was developed to allow for an individual’s conditioning to be gradually built up and did not target a specific heart rate maximum as each person has a different threshold to this form of training. All Adrenaline HIT™ classes were taught by certified gym instructors. Before commencing Adrenaline HIT™ classes participants were required to attend a functional training assessment with a certified trainer to introduce all the Adrenaline HIT™ workout formats. Here they also received appropriate instruction on form and/or were offered modifications as required. Again participants were urged to only do what was comfortable and to not push themselves beyond their own physical limits. Each participant was entered into the membership database of their gym of choice at check-in, before each class, so that attendance and compliance could be accurately tracked.

Much of the previous yoga research has been conducted either with no control group, or using a waitlist control group, consequently there has been a call for future studies to utilize an alternate physical activity intervention as the control to allow for more direct comparisons [48]. High intensity interval training has been specifically chosen as the comparison exercise because it is also vigorous in nature, and in line with endorsed exercises for testing and prescription [49]. That is, programs that incorporate individual tailoring, supervision, stretching, and strengthening and are associated with the best outcomes [50]. Adrenaline HIT™, specifically, is designed around being scaled to individual fitness levels. This is important, especially since individuals who suffer from a persistent pain condition and/or have a history of trauma have a high incidence of comorbidity, and therefore both Bikram yoga and Adrenaline HIT™, are modifiable to accommodate individual abilities. Furthermore, both the Bikram yoga and the Adrenaline HIT™ classes are standardized, commonly available and offered at multiple sites around Melbourne, at different class times, to maximize the potential for attendance. Lastly, Adrenaline HIT™ classes were also anticipated to provide similar benefits of in-group social gains as the Bikram yoga classes [51].

## Measures

### Screening

Prior to recruitment, participants completed the MINI International Neuropsychiatric Interview as a part of the screening protocol. The MINI is a brief structured face to face interview schedule for the major Axis 1 psychiatric disorders for the Diagnostic and Statistical Manual of Mental Disorders, Fourth Edition (DSM-IV) [52]. This was done to screen for those individuals that might psychologically adversely affected due to participation.

### Primary outcome measure

The primary outcome measure was pain (totalled score of severity plus interference [BPI TOT]) as measured by the BPI. The BPI provides a severity rating on a scale of 0-no pain to 10-pain as bad as you can imagine for current pain severity and for pain severity over the last 24 h at its worst, at its least and at its average. As well as an interference score across 7 domains of quality of life ratings, on a scale of 0-does not interfere to 10-interferes completely. Both intensity and interference scales were scored as the means of the four and seven items respectively [36, 53]. The minimum clinically important difference (MCID) for the BPI has been reported as an ~ 2 point difference in average severity scores [37]. The BPI has been shown to have an internal consistency α = .85 for the intensity items and α = .88 for the inference items for chronic non-malignant pain [53] and is one of the instruments recommended by the Initiative on Methods, Measurement, and Pain Assessment in Clinical Trials group [54].

### Secondary exploratory outcome measures

A battery of self-report questionnaires was completed online by participants in the week before commencing their intervention and again in the week after completing their intervention. These comprised the:

i) Medical Outcomes Study Short Form 36 Health Survey (SF-36). The SF-36 is a short form (36 questions) generic health survey providing 8 profile scores (physical functioning, role physical, general health, body pain, role emotional, social functioning, vitality and mental health) along with physical and mental health summary scores, all of which range from 0 to 100. Questions were asked around a participant’s present experience or during the past 4 weeks [55, 56].. The SF-36 reliability has been assessed in more than 25 studies with Cronbach’s alpha usually exceeding α = 0.80 [56] and it has been psychometrically tested within the Australian population [55].
ii) Depression, Anxiety and Stress Scale (DASS-21). The DASS-21 provides a measure of general negative affective syndromes and consists of 21 negative emotional symptoms across depression, anxiety and stress (7 items each). The experience of each symptom over the past week is rated on a 4-point severity/frequency scale [57]. Scores were determined by summing the scores for the relevant 7 items [58]. Internal consistencies for each of the scales for the DASS-21 were: Depression α = 0.94; Anxiety α = 0.87; Stress α = 0.91 [58].
iii) Self-report Inventory for Disorders of Extreme Stress (SIDES-SR). The SIDES-SR is a self-report measure that assesses the six dimensions of impairment in Disorders of Extreme Stress [59]. These are: affect regulation, amnesia and dissociation, somatization, disruptions in self-perception, disorders in relationships with others, and disrupted systems of meaning. For each item participants were required to indicate yes/no for lifetime presence, and also to rate the current symptom presence and severity during the past month from 0 to 3. The overall SIDES-SR has been shown to have a high internal consistency (α = .93) [60].
iv) The Coping Self –Efficacy scale (CSE). The CSE scale provides a measure of a person’s perceived ability to cope effectively with life challenges, as well as a way to assess changes in CSE over time in intervention research. Items are rated on an 11-point scale the extent to which they believe they could perform behaviours important to adaptive coping and consists of 26 items where an overall score is created by summing the item ratings [61]. The CSE has an internal consistency of α = 0.95 [61].
v) Life Stressor Checklist-Revised (LSC-R). LSC-R is a self-report measure that assesses traumatic and stressful experience across 30 life events, for example, death of a relative, natural disasters, and physical or sexual assault. If an event was endorsed then they were asked if they were still being impacted by it in the last 12 months (yes or no), and if so, asked to rate how much using a five-point intensity scale (1- not at all, to 5-extremely) [42]. The LSC-R has a test–retest reliability Kappa values range of 0.52–0.97 across the life events and good concurrent validity with the Symptom Checklist-90-Revised (SCL-90-R) and the Impact of Event Scale-Revised (IES-R) [62]
vi) Five Facet Mindfulness Questionnaire (FFMQ). The FFMQ is a 39-item self-report assessing multiple facets of mindfulness. Participants’ were required to rate each item on a 5-point scale. The five main facets representing mindfulness are: observing, describing, acting with awareness, non-judging of inner experience and non-reactivity to inner experience and combining the five factors provides a measure of multidimensional mindfulness as a whole [63]. The FFMQ has been shown to have good internal consistency [63].

Participants were also required to attend a physiological testing appointment at our lab. During the testing appointment participants’ height, weight, and waist/neck/hip circumferences were measured. Also, participants underwent a resting ECG assessment and blood pressure test. The ECG data was used to extract measures of Heart Rate Variability (HRV), specifically, the Standard Deviation of the Normal beat to Normal beat interval (SDNN), Low Frequency normalised units (LFnu) and High Frequency normalised units (HFnu). These measures of HRV have been previously associated with chronic pain levels [64].

### Sample size and randomization

Sample size was determined bearing in mind recruitment, methodology and funding feasibility [65]. A 26% dropout rate was assumed based on a previous study that had utilized Bikram yoga [66]. It was estimated that a total sample size of 60 participants, randomized across the Bikram yoga and HIIT interventions would provide 80% power to demonstrate a minimum clinically important change in BPI total pain scores of 2.0 [37].

After obtaining written consent, the participants were randomized in a 1:1 ratio to Bikram Yoga or HIIT using a random number table (consisting of 1’s and 2’s) generated in Microsoft Excel (2010). Participants were asked to continue with whatever concurrent drug and psychotherapies they were already undertaking but not to stop or start anything new while participating. Allocation to intervention or control took place at recruitment using the random number table which was maintained by a researcher blinded to the treatment allocations (blinded to which activity was 1 and which activity was 2). This was an open-label study as participants were aware of the intervention they were in, however, both arms of the intervention were presented as the experimental arm; knowledge of group assignment for the participants, and the instructors, were expected to impact participation similarly. Randomization methodology followed the low risk bias criteria outlined in The Cochrane Collaboration protocol for ‘Yoga treatment for chronic non-specific low-back pain’ [67].

### Data analysis

We conducted an intention-to-treat analysis, such that, all participants were encouraged to complete t1 data collection regardless of their level of compliance with the interventions. Additionally, all descriptive statistics reported have been calculated without replacing missing data while single imputation of the mean was used to replace missing data in the multivariate analyses. Also in the multivariate analyses, square-root transformations were performed for the variables that had outliers affecting both the assumptions and results [68]. All statistical analyses were conducted using IBM SPSS Statistics for Windows, Version 24.0 (Armonk, NY: IBM Corp), and ECG output was analysed using LabChart 8.0 Pro software (ADInstuments, Australia).

Repeated Measures ANCOVA analysis was used to assess the primary outcome, that is, whether post intervention pain levels, adjusted for the pre-test scores, significantly differed between groups. This allowed for a direct comparison of treatment efficacy between the two groups. Repeated Measures ANCOVA was also used to assess the impact of the interventions on the remaining measures as per the secondary aims.

Effect size for each measure was calculated to estimate the variability between groups and also estimate the required sample size of a larger study for a significant result to be detected. Finally, to assess the feasibility of conducting a vigorous exercise intervention among people with persistent pain, the number of classes attended per week by the participants and study and group intervention adherence levels were monitored.

A repeated measure ANOVA of the primary outcome was used to assess whether participants’ BPI TOT pain reduced from pre to post intervention. And finally t1 minus t0 change descriptive statistics (with standard error of the mean confidence intervals) were plotted to illustrate trends of change for all measures.

## Results

### Descriptive statistics

**Fig. 2:**
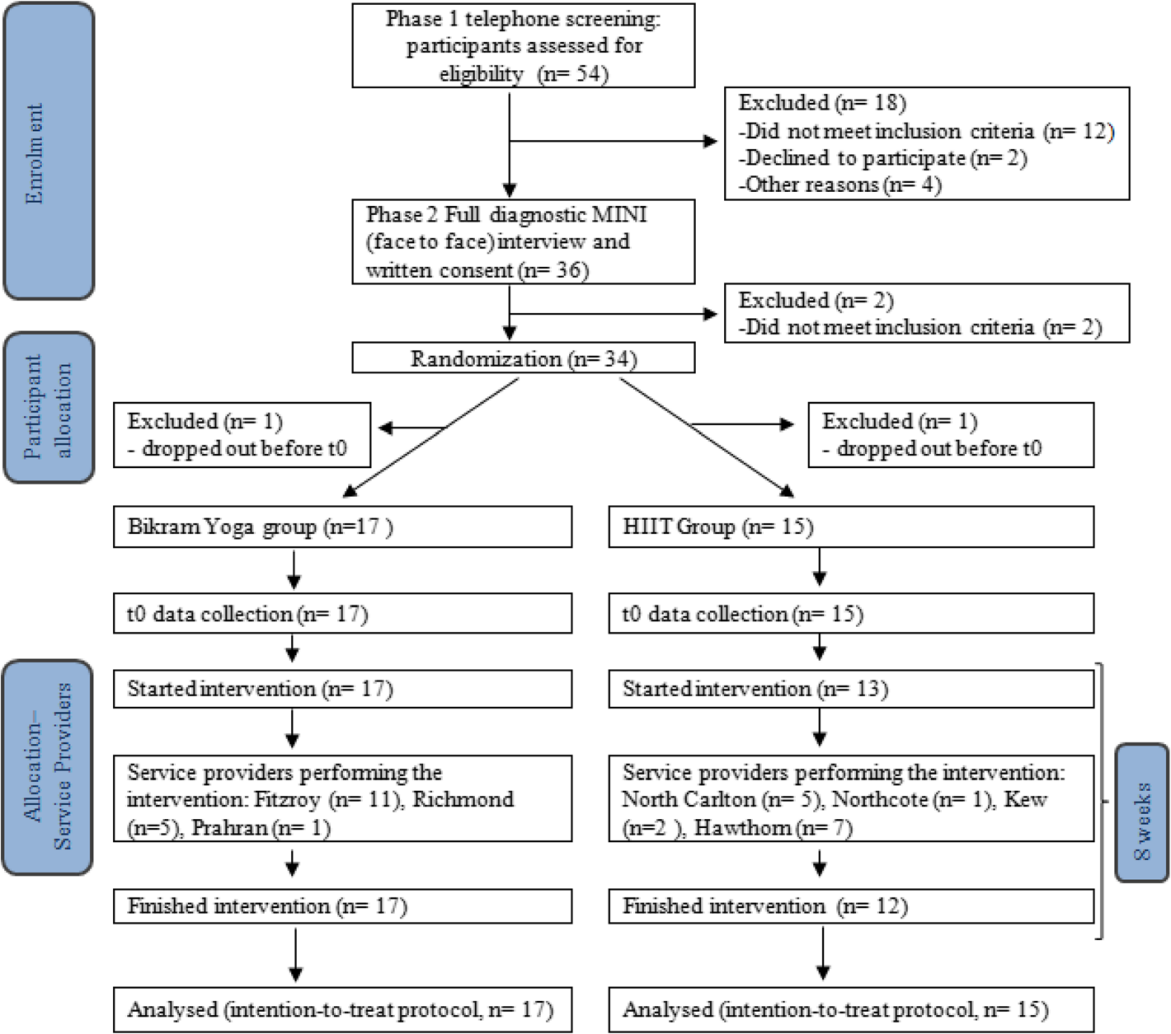
Participant study flow diagram adapted from the CONSORT RCT flow diagram

Thirty-four women were recruited for the study; however, two women were excluded from analysis as they dropped out after randomization and before t0 data collection. Of the remaining thirty-two: 17 women were randomized to the Bikram yoga intervention and 15 women to the HIIT control group; the study’s participant flow diagram is presented in Fig. 2. The womens’ ages ranged from 21 to 47 years with an average age of 30.2 years (Table 2). Over 50% of participants had completed tertiary studies and differences in body mass index and hip to waist ratio between the two groups were negligible at baseline. Twenty-eight of the thirty –two women had a formal persistent pain diagnosis (Table 3). The most common pain condition amongst the women was fibromyalgia (22.4%), followed by lower back pain (15.6%), chronic pelvic pain (12.8), back and neck pain (9.4%), and chronic widespread pain (9.4%). Other persistent pain conditions included sciatica, inflammatory arthritis, joint, musculoskeletal, back, and foot pain, temporomandibular joint disorder, and patella femoral pain syndrome (Table 3). Participants reported taking various medications and were instructed to keep taking their medications as usual as medication change was not part of this study.

**Table 2:** Participant characteristics and demographics in total, and within each group (Bikram yoga and HIIT)

	Total (*N* = 32)			Bikram (*n* = 17)			HIIT (*n* = 15)		
	M	SD	Range	M	SD	Range	M	SD	Range
Age (years)	30.2	8	21–47	31.1	7.4	21–45	29.2	8.9	21–47
Height (cm)	167.8	6.3	156–181	166.5	4.4	160–174	169.2	7.8	156–181
Weight (kg)	75.8	15.4	48–105	75.9	13.7	53–105	75.7	17.6	48–103
BMI	26.9	5.4	19.1–37.2	27.4	5.0	19.7–37.2	26.4	5.9	19.1–37.2
Waist	84.6	14.1	64–112	86.4	12.8	69–112	82.6	15.5	64–106
Hip	106.5	10.8	89–127	106.7	9.9	90–124	106.3	12.0	89–127
Waist: Hip ratio	0.8	0.1	0.6–1.0	0.8	0.1	0.6–1.0	0.8	0.1	0.6–0.9
Average number of classes attended per week	1.3	0.9	0.0–2.9	1.4	0.9	0.1–2.9	1.2	0.9	0.0–2.25
Highest level of education, n %									
No formal education	0	na	0		na		0	na	
Year 10 or equivalent	0	na	0		na		0	na	
Tear 12 or equivalent	6	18.8	2		11.8		4	26.7	
Trade/Apprenticeship	1	3.1	1		5.9				
Certificate/Diploma	5	15.6	3		17.6		2	13.3	
University Degree	9	28.1	4		23.5		5	33.3	
Higher University Degree	11	34.4	7		41.2		4	26.7	
Trauma history, number and % of participants reporting an event (still impacting their life in the last 12 months)									
Physical trauma events (only)	6	18.8		4	23.5		2	13.3	
Psychological trauma events (only)	29	90.1		16	94.1		13	86.7	
Events with a combined physical and psychological impact	18	56.3		9	52.9		9	60.0	
Total number of times a trauma event was reported as still impacting lives in the last 12 months (LSC-R-tot) and the range of the number of events per participant									
	LSC-R-tot	Range		LSC-R-tot	Range		LSC-R-tot	Range	
Physical trauma events (only)	7	1–2		6	1–2		1	1–1	
Psychological trauma events (only)	94	1–9		52	1–8		42	1–9	
Events with a combined physical and psychological impact	57	1–11		27	1–8		30	1–11	

Abbreviations: *HIIT* High Intensity Interval Training, *M* Mean, *SD* Standard Deviation, *cm* Centimeters, *kg* Kilograms, *BMI* Body mass index, LSC-R-tot total number of Life Stressor Checklist Revised trauma events participants’ reported still being impacted by in the last 12 months. Previous trauma events were categorized into events with physical impacts only, events with psychological impacts only and events with a combined physical and psychological impact

**Table 3:** Persistent pain diagnosis and conditions of the sample

	Total (*N* = 32)		Bikram (*n* = 17)		HIIT (*n* = 15)	
	*n*	%	*n*	%	*n*	%
Persistent pain diagnosis						
Yes	28	98.5	14	43.8	14	43.8
No	4	12.5	2	6.3	2	6.3
Persistent pain						
Fibromyalgia	7	22.4	5	15.6	2	6.3
Lower back pain	5	15.6	1	3.2	4	12.5
Chronic pelvic pain	4	12.8	2	6.3	2	6.3
Back and neck pain	3	9.4	2	6.3	1	3.2
Chronic widespread pain	3	9.4	1	3.2	2	6.3
Sciatica	1	3.2	1	3.2	0	na
Inflammatory arthritis	1	3.2	0	Na	1	3.2
Joint pain	1	3.2	1	3.2	0	na
Musculoskeletal pain	1	3.2	1	3.2	0	na
Temporomandibular disorder	1	3.2	0	0	1	3.2
Patella femoral pain syndrome	1	3.2	0	na	1	3.2
Foot pain	1	3.2	1	3.2	0	na
Neck pain	1	3.2	1	3.2	0	na
Back pain	1	3.2	1	3.2	0	na
Knee pain	1	3.2	0	na	1	3.2

At baseline, many participants continued to be impacted by a past trauma event and many reported having multiple trauma events (Table 2). Over 90% of women in this study reported still being impacted in the last 12 months by a previous psychological trauma, 56.3% by a previous combined physical and psychological trauma, and less than 20% of the women reported still being impacted by a past physical trauma. Furthermore, many of the women reported multiple trauma events; for example, one participant reported still being impacted by 11 separate past trauma events of a combined physical and psychological nature. Also at baseline, higher BPI TOT scores were seen to correlate with poorer SF-36 quality of life scores, a greater number of past impactful trauma events, and a higher heart rate (Additional file 1: Table S1).

### Between group t1 differences with effect sizes

The Analysis of Covariance (ANCOVA) analysis that statistically compared differences between the Bikram yoga and the HIIT groups’ t1 scores, while accounting for t0 scores, revealed no significant difference between the two groups on the primary outcome measure of pain (BPI TOT), F(1, 29) = 0.01, *p* = 0.000 (Table 4). Furthermore, the results showed no trend that Bikram yoga had a better pain outcome.

**Table 4:** ANCOVA comparison of Bikram yoga versus HIIT scores of primary and secondary outcomes at post-intervention (t1) after controlling for baseline (t0) scores. Displayed are t0 means (M) and standard deviations (SD), ANCOVA t1 estimated marginal means (M), SD, F value, *p*- value, and partial eta-squared effect sizes. Missing data was replaced by single imputation of the mean

	t0 Bikram yoga (*n* = 17)		t0 HIIT (*n* = 15)		t1 Bikram yoga (*n* = 17)		t1 HIIT (*n* = 15				Partial eta squared
Response Variable	M	SD	M	SD	M^a^	SD	M^a^	SD	*F*-value	*p*-value	
Primary Outcome											
BPI TOT	6.80	3.16	9.23	3.79	6.79	3.71	6.67	4.16	0.01	0.914	0.000
BPI-Severity	3.23	1.50	4.61	1.43	3.19	1.52	3.47	1.86	0.32	0.577	0.011
BPI-Interference	3.57	1.84	4.41	2.49	3.44	2.48	3.37	2.41	0.01	0.920	0.000
Secondary Outcomes											
SF-36 PF	74.41	16.85	72.67	20.34	80.91	18.27	68.96	18.45	6.17	0.019*	0.175
SF-36 RP	52.21	23.79	56.67	25.92	54.90	18.85	54.13	23.52	0.01	0.918	0.000
SF-36 BP	45.94	15.40	42.67	20.61	55.39	18.60	44.75	21.21	2.35	0.136	0.075
SF-36 GH	36.66	19.97	23.36	20.80	44.85	21.29	43.51	23.55	0.08	0.786	0.003
SF-36 VT	30.15	22.99	29.17	18.85	39.13	20.58	39.29	15.87	0.001	0.978	0.000
SF-36 SF	52.21	23.90	50.00	25.88	64.42	26.23	52.41	23.07	1.96	0.173	0.063
SF-36 RE	53.43	29.03	48.33	27.13	66.12	23.84	65.77	25.42	0.02	0.965	0.000
SF-36 MH	54.12	21.16	47.00	19.07	63.94	16.67	49.37	16.42	9.09	0.005*	0.239
DASS-21 Stress	10.29	4.48	12.00	3.91	8.93	5.42	9.52	4.52	0.11	0.742	0.004
DASS-21 Anxiety	5.94	4.60	7.47	4.93	5.16	4.18	6.16	6.12	0.50	0.484	0.017
DASS-21 Depression	8.76	5.67	9.27	5.06	6.69	5.78	7.38	6.30	0.12	0.732	0.004
SIDES-SR total	12.78	9.87	15.46	12.88	10.03	9.10	10.79	10.72	0.07	0.795	0.002
FFMQ total	117.41	19.94	110.20	50.42	120.41	13.94	122.47	19.93	0.28	0.600	0.010
CSE	123.76	45.91	115.07	20.94	142.45	46.53	130.66	50.24	0.59	0.450	0.020
HR	67	12	72	12	69	10	72	10	0.82	0.374	0.028
SDNN	65.27	33.09	53.11	18.14	68.01	36.43	51.94	14.75	5.61^2^	0.025*^b^	0.162
LFnu	48.99	16.21	49.75	18.21	44.94	23.76	55.33	17.85	2.94	0.097	0.092
HFnu	47.17	15.65	48.08	16.73	54.02	22.14	42.59	16.93	3.49	0.072	0.107
SAP	112	9	109	9	112	11	112	8	0.02	0.905	0.001
DAP	73	11	71	10	74	10	74	10	0.10	0.755	0.003

Abbreviations: *HIIT* High Intensity Interval Training, *SD* Standard deviation, BPI TOT combined severity and interference scores of the Brief Pain Inventory, *PF* Physical Functioning, *RP* Role Physical, *BP* Body Pain, *GH* General Health, *VT* Vitality, *SF* Soc*i*al Functioning, *RE* Role Emotional, *MH* Mental Health, *DASS* Depression, Anxiety, and Stress Scale, *SIDES-SR* Self-Report Instrument for Disorders of Extreme Stress, *FFMQ* Five Factor Mindfulness Scale, *CSE* Coping Self-Efficacy Scale, *HR* Heart Rate, *HRV* Heart Rate Variability, *SDNN* Standard Deviation of the Normal beat to Normal beat interval, *LFnu* Low-Frequency normalized units, *HFnu* High-Frequency normalized units, *SAP* Systolic arterial pressure, *DAP* Diastolic arterial pressure

*Significant: *p* < 0.05

^a^ ANCOVA estimated marginal means (adjusted for t0 scores)

^b^A square-root transformation adjusted to remove outliers

Partial eta squared: small effect (0.01) - medium effect (0.09), large effect (0.25)

Further ANCOVA analyses comparing t1 score differences in the secondary exploratory outcome measures (Table 4) revealed statistically significant improvements between the groups for the SF-36 physical functioning subscale, F(1, 29) = 6.17, *p* = .019, and partial eta-squared effect size (η_p_^2^) = .175. The estimated marginal means showed that the Bikram yoga group had greater improvement in physical functioning (*M* = 80.91), compared to HIIT (M = 68.96). Also, a statistically significant improvement between the groups was seen for SF-36 mental health subscale, F(1, 29) = 9.09, *p* = .005, η_p_^2^ = .239. Estimated marginal means showed that the Bikram yoga group had greater improvement in mental health (M = 63.94) compared to HIIT (M = 49.37). Lastly, a statistically significant improvement between the groups was seen for the heart rate variability measure of SDNN, F(1, 29) = 5.12, *p* = .013, η_p_^2^ = .150. Estimated marginal means showed that the Bikram yoga group had increased SDNN (M = 68.01) compared to HIIT(M = 51.94). Furthermore, the medium η_p_^2^ found for the SF-36 physical functioning subscale and heart rate variability (SDNN), and the moderate to large effect of group found for the SF-36 mental health subscale, all in favor of Bikram yoga, may be of some use in future study designs.

### Feasibility analysis

In this study, class attendance, participant retention and the change in t1 minus t0 BPI TOT scores suggest it was feasible to conduct a vigorous exercise intervention among people with persistent pain. The average number of classes per week attended by the women during the intervention was 1.3 (SD = 0.9, range = 0 to 2.9), with no significant difference found between attendance in the Bikram Yoga group compared to the HIIT group (Table 2). Overall, 12 participants attended between 0 to 8 classes (Bikram yoga = 6, HIIT = 6), 14 attended between 9 and 16 classes (Bikram yoga =7, HIIT = 7), and 6 participants attended between 17 and 24 classes (Bikram yoga = 4, HIIT = 2). The overall an intention-to-treat analysis completion rate of the women was 91%; 100% (17 out of 17) in the Bikram yoga group and 80% (12 out of 15) in the HIIT group.

Finally, a repeated measures ANOVA, comparing t1 minus t0 scores indicated a reduction in BPI TOT pain levels. Given the previous BPI TOT ANCOVA revealed that the t1-t0 BPI TOT difference between the Bikram yoga and HIIT groups was negligible, this analysis was conducted considering the total sample as one group. BPI TOT scores between t1 (M = 6.75, SD = 3.98) and t0 (M = 7.94, SD = 3.63) were found to significantly reduce, F(1, 31) = 6.29, *p* = 0.018, partial eta squared = 0.169.

### Safety analysis

**Fig. 3:**
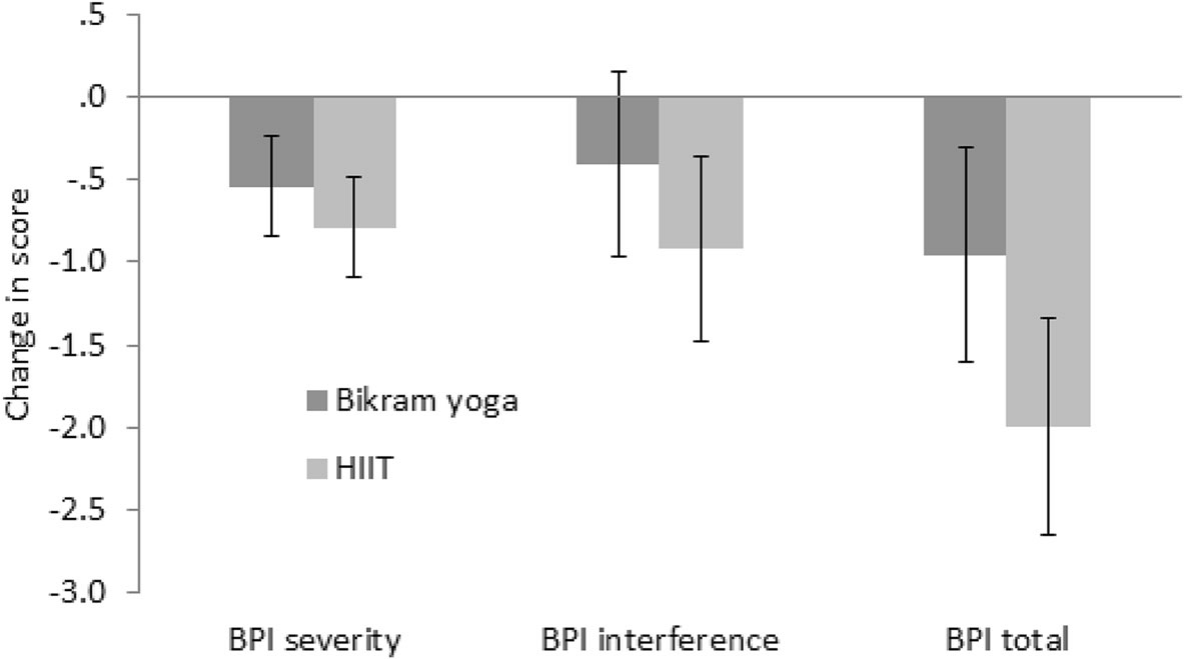
Change (t1 minus t0) descriptive (mean and standard error of the mean confidence intervals) comparisons of Brief Pain Inventory (BPI), total, severity, and interference scores for Bikram yoga (*n* = 17) versus the High Intensity Interval Training (HIIT) (*n* = 12) groups. Change statistics were calculated without replacing missing data

As above, the repeated-measures Analysis of Variance (ANOVA), comparing t1 minus t0 scores indicated a significant reduction in BPI TOT pain levels for the group as a whole (Fig. 3), with the t1minus t0 BPI TOT difference between the Bikram yoga and HIIT groups being negligible (Table 3). However, while overall there was trend of improvement in pain, when considering individual changes in average BPI severity scores, of the 32 women: 19 reported a lower average BPI severity score at t1 compared with t0 (5 with decreases greater than the MCID of ~ 2, 7 with decreases between 1 and 2, and 7 with decreases between 0 and 1); 2 had zero change in BPI severity scores; and 8 reported a higher average BPI severity score (1 with an increase of 2, and 7 with increases between 0 and 1).

Nevertheless, no intervention related injuries were reported by participants. Of those that had to stop their prescribed program, one participant found the delivery of the HIIT instructions to be overwhelming, one found the level of post-class muscle soreness and recovery time to be too great (HIIT), and one found the exercise too triggering of their persistent pain that suggested modifications were unable to resolve (Bikram yoga). Other reasons for low class attendance rates were absence due to cold and flu, injury sustained during the 8-weeks extraneous to the study, and unforseen short trips interstate and overseas.

**Fig. 4:**
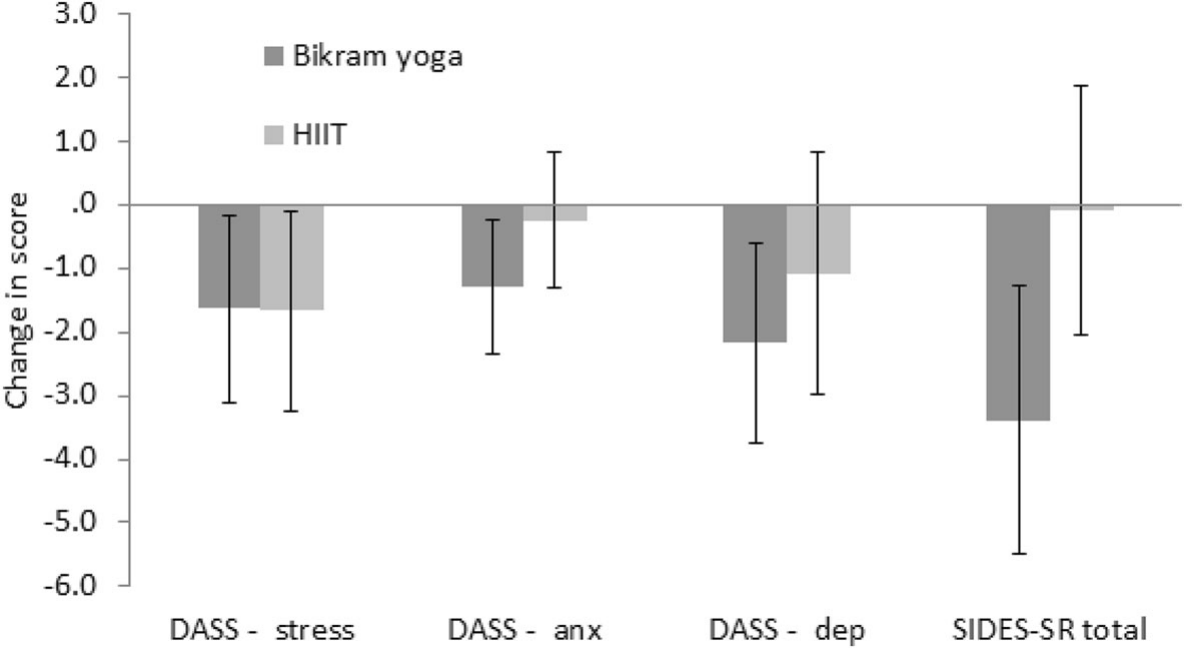
Change (t1 minus t0) descriptive (mean and standard error of the mean confidence intervals) comparisons of Depression (dep), Anxiety (anx) and Stress Scale (DASS-21), and Self-Report Inventory of Disorders of Extreme Stress (SIDES-SR) scores for Bikram yoga (*n* = 17) versus the High Intensity Interval Training (HIIT) (*n* = 12) groups. Change statistics were calculated without replacing missing data

**Fig. 5:**
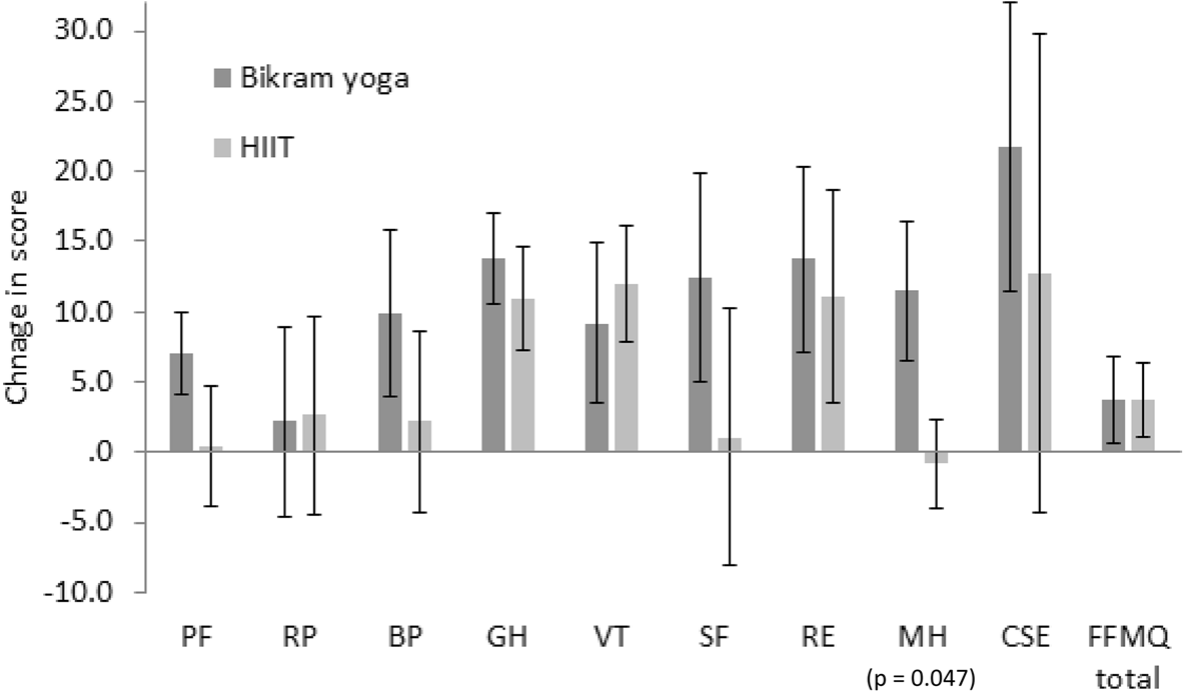
Change (t1 minus t0) descriptive (mean and standard error of the mean confidence intervals) comparisons of SF-36: Physical Functioning (PF), Role Physical (RP), Body Pain (BP), General Health (GH), Vitality (VT), Social Functioning (SF), Role Emotional (RE), Mental Health (MH), and Coping Self-Efficacy (CSE), and Five Factor Mindfulness Questionnaire (FFMQ) scores for Bikram yoga (*n* = 17) versus the High Intensity Interval Training (HIIT) (*n* = 12) groups. Change statistics were calculated without replacing missing data. *P*-values are shown for measures where the difference of change seen between the groups was found to be significant

**Fig. 6:**
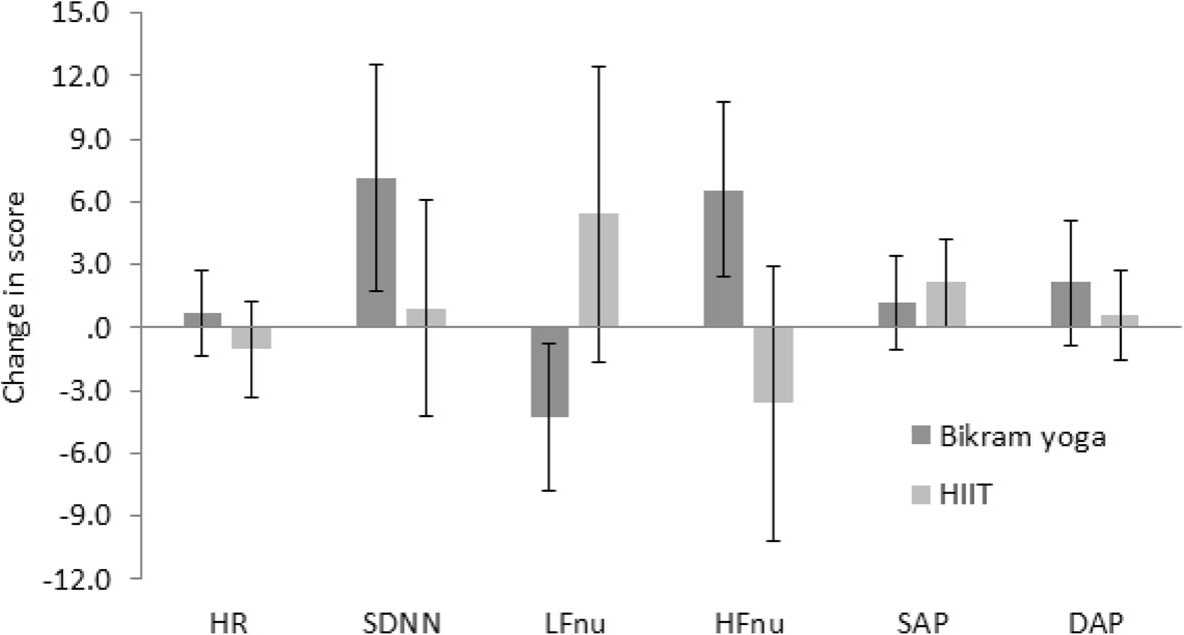
Change (t1 minus t0) descriptive (mean and standard error of the mean) comparisons of Haemodynamic (Heart Rate [HR], Systolic Blood Pressure [SAP], Diastolic Blood Pressure [DAP]) and heart rate variability (Standard Deviation of the Normal beat to Normal beat interval [SDNN], Low-Frequency normalised units [LFnu], High-Frequency normalized units [HFnu] measures for Bikram yoga (*n* = 17) versus the High Intensity Interval Training (HIIT) (*n* = 10) groups. Change statistics were calculated without replacing missing data

Many of the pre to post-intervention changes in outcome scores demonstrated improvement (Figs 3, 4, 5 and 6), and independent sample t-tests were conducted on the change statistics but are only to be regarded as descriptive statistics for feasibility purposes as none of the missing data has been replaced. Interestingly, although no significant group differences were found between the groups for the change in scores for any of the physiological measures, the pattern of change for heart rate variability measures of LFnu and HFnu between the groups was opposite. That is, LFnu levels in the Bikram yoga group dropped comparable to the level of HFnu drop seen in the HIIT group and vice versa; LFnu increased in the HIIT group at a similar level of increase to that of HFnu in the Bikram yoga group (Fig. 6).

## Discussion

The present study found no difference in BPI TOT scores between women participating in a course of Bikram yoga compared with those completing a course of HIIT. Consequently, these results can not inform power calculations of future pain studies. However, as a whole, the women in this study did achieve statistically significant improvement in persistent pain levels, although it is impossible to determine if these improvements were specific to vigorous exercise. While no difference was seen between the two types of exercise in relation to pain, the Bikram yoga group achieved significant improvements in self-reported measures of physical functioning, mental health, and a physiological measure of heart rate variability, compared with the HIIT group. Furthermore, overall pain levels were diminished, only 3 women (9%) stopped attending classes after finding them too intense to continue, and the intention-to-treat completion rates were high. The results of the present study suggest that vigorous exercise interventions might be a feasible undertaking for people with a persistent pain condition.

Across the entire sample, the primary outcome measure (BPI TOT) was seen to significantly decrease from t0 to t1 but the level of decrease seen between the two types of vigorous exercise was not significant. As a result, it is not appropriate to conclude that the improvement in pain levels was due to the vigorous exercise as there are other influences that might also positively impact pain levels. For example: the Hawthorne effect [69]; physical activity and exercise (not vigorous) [70]; and the social benefits of participating in group exercise classes [71] may have all contributed to the overall positive pain outcomes. Future research might involve a waitlist control arm and a non-vigorous exercise intervention arm to help differentiate whether any improvements in persistent pain levels can be attributed to the undertaking of a vigorous exercise intervention, and the waitlist control would differentiate from any social group-exercise and Hawthorne related trial effects.

The Bikram yoga group did see significant increases in the SF-36 physical functioning subscale scores, SF-36 mental health subscale scores, and the SDNN measure of heart rate variability, compared with the HIIT group. SF-36 physical functioning and mental health represent a physical and a mental assessment of quality of life. Improved quality of life outcomes have been frequently associated with a reduced burden of disease, most commonly arthritis, back pain, depression, diabetes, and hypertension [72]. Increased SDNN is associated with increased heart rate variability which is considered a physiological indicator of better health [73]. Specifically, increased heart rate variability is an indicator of improved parasympathetic functioning [74] which has been associated with a wide range of positive medical [75, 76] and psychological [77, 78] health outcomes. Conversely, decreased heart rate variability has been associated with increased mortality [79]. With regards to pain specifically, a meta-analysis of 11 studies investigating associations between persistent pain and heart rate variability, reported a large significant effect of decreased SDNN with increased persistent pain (− 5.43 (95% CI [− 8.54 to − 2.32]) [64].

While our hypothesis that Bikram yoga would be the superior vigorous exercise intervention for improving pain was not supported, the above results provide limited evidence that Bikram yoga may have potential for targeting psychobiological mechanisms such as improved autonomic dysregulation and decreased allostatic load. For example, increased SDNN heart rate variability has been associated with improved sympatho-vagal balance [80]. And lower burden of disease has been associated with less allostatic load [81], specifically with regard to physical functioning [82] and mental health [83]. Therefore, Bikram yoga may have potential as a bottom-up, sensorimotor exposure therapy to improve autonomic dysregulation, and decrease allostatic load, but more evidence is needed.

The effect sizes were consistent with the between group differences observed, therefore, the outcome of the present study can be used to estimate required sample size for future studies investigating the efficacy of Bikram yoga for improving quality of life, physical functioning, mental health, and physiological indicators of better health such as heart rate variability. Clearly any intervention related change in scores must still be viewed cautiously due to the potential bias of placebo and Hawthorn effects. For example, it has previously been reported that the placebo effect accounts for approximately half of any improvements in the self-report psychological measures in exercise interventions [84]. However, the SDNN measure of heart rate variability is a physiological measures obtained via an ECG assessment. Although this was the only physiological measure to see such a change, a previous yoga study has also reported a significant change in HRV, with no change in other ECG measures [85]. Consequently, arguments that the positive gains of the study are bias due to the placebo effect may not be entirely valid. Furthermore, bias in the differences between the groups due to the Hawthorne effect [86] might also be considered minimal as reportedly, the Hawthorne effect varies depending on the level of participation [87]. In the present study, both the experimental and control arms had similar levels of participation.

Our findings suggest that vigorous exercise interventions may be safely undertaken by selected individuals with persistent pain. Firstly, on average, persistent pain levels were not seen to worsen. Secondly, the retention rates, overall and per group, were acceptable for a persistent pain exercise intervention [88, 89]. However, the prescribed number of classes per week was three but the average number of classes attended each week was 1.4 for the Bikram yoga group and 1.2 for the HIIT group. This suggests that 3 classes per week was too much for the participants, perhaps due to such things as physical and muscle recovery time [90]. Future vigorous exercise interventions involving persistent pain participants could decrease the prescribed exercise dose, to two classes per week for example, and they would still adhere to the Delphi recommendations [48].

The study had several limitations. The recruited participants were women aged 20 to 50, self-selected volunteers from the community. They were informed at recruitment about the vigorous nature of the intervention and only those who felt they would be able to manage such intensive exercise proceeded. This is the main reason why one third of the 54 women who inquired about the study did not proceed. Therefore, the feasibility of a vigorous exercise intervention for persistent pain is biased to those who feel high functioning enough to manage it. Also, participants were not exposed gradually to the exercises however, both exercises lend themselves for the participants to be able to go at their own pace. Instead, participants were encouraged to attend three classes from the beginning. Intensity was increased with each class by the participant at their pace. Another limitation was the 8-week length of the intervention as this is the minimum recommended Delphi dose [48]. The better quality studies (rated using the Oxford Level of Evidence) identified in a number of systematic reviews of yoga for pain [30, 91–93], all had intervention lengths of between 12 weeks [94–96] and 24 weeks [97]. Eight weeks may not be a sufficient length of time for yoga to impact pain and any future Bikram yoga for pain studies should be at least 12 weeks long. Furthermore, although the Bikram yoga group saw significant improvements in mental health, physical functioning and heart rate variability, as compared to the HIIT group, this study was not powered on such measures. Additionally, there was no adjustment for multiple analyses within the secondary outcome measures. Therefore the significance of these results should be viewed with caution.

Lastly, even though no one participating in this study suffered any injuries due to taking part, some adverse effects were seen. However, the rates of adverse events were not greater than those previously reported by less vigorous yoga intervention studies [91]. Also, a few participants who didn’t complete didn’t ever start their exercise classes. These were all assigned to the HIIT group and the process of getting the participants enrolled into the gyms conducting the HIIT classes was longer and more complicated that it was for the Bikram yoga studios. It is believed this may have impacted the momentum of participation and we suggest that future studies need to make access to interventions as streamlined as possible.

## Conclusions

In conclusion, women with persistent pain and a history of trauma undertaking an 8-week, 3 class per week vigorous exercise intervention reported improved levels of persistent pain. As no differences in pain levels between the Bikram yoga and the HIIT group were seen, no conclusions about the efficacy of Bikram yoga compared with vigorous exercise for persistent pain can be made. However, the imporvements in self-report quality of life measures and a physiological indicator of better health, seen in the Bikram yoga group as compared to HIIT, suggest there may be other health-related benefits to the Biikram yoga practice that warrant further exploration. Lastly, the outcomes of the present study suggest vigorous exercise interventions invloving people with persistent pain might be feasible.

## Additional file

Additional file 1: **Table S1.** Total sample (*N* = 32) baseline means (M) and standard deviations (SD) of primary and secondary outcome measures and correlations with persistent pain levels (Brief Pain Inventory Total (BPI TOT]) (DOCX 19 kb)

## Data Availability

The datasets used and/or analysed during the current study are available from the corresponding author on reasonable request.

## Abbreviations

ACTRN: Australian New Zealand Clinical Trials Registry
ANCOVA: Analysis of Covariance
ANOVA: Analysis of Variance
BPI TOT: Totalled BPI severity and interference scores
BPI: Brief Pain Inventory
CSE: Coping Self –Efficacy scale
DASS-21: Depression, Anxiety and Stress Scale
DSM: Diagnostic and Statistical Manual of Mental Disorders
FFMQ: Five Facet Mindfulness Questionnaire
HFnu: High Frequency normalised units
HIIT: High Intensity Interval Training
HRV: Heart Rate Variability
IASP: International Association for the Study of Pain
IES-R: Impact of Event Scale-Revised
LFnu: Low Frequency normalised units
LSC-R: Life Stressor Checklist-Revised
M: Mean
MCID: Minimum Clinically Important Difference
MINI: MINI International Neuropsychiatric Interview
PTSD: Post-traumatic Stress Disorder
RCT: Randomized Controlled Trial
SCL-90-R: Symptom Checklist-90-Revised
SD: Standard Deviation
SDNN: Standard Deviation of the Normal beat to Normal beat interval
SF-36: Short Form (36 item) of the Medical Outcomes Scale
SIDES-SR: Self-report Inventory for Disorders of Extreme Stress
t0: Pre-intervention
t1: Post-intervention
η_p_^2^: Partial Eta-squared Effect Size

## References

[1] Gaikwad M, Vanlint S, Moseley GL, Mittinty MN, Stocks N (2017). Understanding patient perspectives on management of their chronic pain–online survey protocol. J Pain Res.

[2] Afari N, Ahumada SM, Wright LJ, Mostoufi S, Golnari G, Reis V, Cuneo JG (2014). Psychological trauma and functional somatic syndromes: a systematic review and meta-analysis. Psychosom Med.

[3] Coker AL, Smith PH, Bethea L, King MR, McKeown RE (2000). Physical health consequences of physical and psychological intimate partner violence. Arch Fam Med.

[4] Paras ML, Murad MH, Chen LP, Goranson EN, Sattler AL, Colbenson KM, Elamin MB, Seime RJ, Prokop LJ, Zirakzadeh A (2009). Sexual abuse and lifetime diagnosis of somatic disorders: a systematic review and meta-analysis. JAMA..

[5] Van Hecke O, Torrance N, Smith B (2013). Chronic pain epidemiology and its clinical relevance. Br J Anaesth.

[6] McFarlane AC (2010). The long-term costs of traumatic stress: intertwined physical and psychological consequences. World Psychiatry.

[7] Morasco BJ, Lovejoy TI, Lu M, Turk DC, Lewis L, Dobscha SK (2013). The relationship between PTSD and chronic pain: mediating role of coping strategies and depression. Pain..

[8] Powers A, Fani N, Pallos A, Stevens J, Ressler KJ, Bradley B (2014). Childhood abuse and the experience of pain in adulthood: the mediating effects of PTSD and emotion dysregulation on pain levels and pain-related functional impairment. Psychosomatics..

[9] Yehuda R, Hoge CW, McFarlane AC, Vermetten E, Lanius RA, Nievergelt CM, Hobfoll SE, Koenen KC, Neylan TC, Hyman SE. Post-traumatic stress disorder. Nat Rev Dis Primers. 2015;1:1–22. 10.1038/nrdp.2015.57 27189040

[10] American Psychiatric Association. Diagnostic and statistical manual of mental disorders, (DSM-5®), 5th Ed. Am Psychiatr Publ. 2013. pp. 271–280.

[11] Arnsten AF (2009). Stress signalling pathways that impair prefrontal cortex structure and function. Na Rev Neuroscience.

[12] McEwen BS, Schulkin J (2004). Protective and damaging effects of the mediators of stress and adaptation: Allostasis and allostatic load. *Allostasis, homeostasis, and the costs of physiological adaptation*.

[13] McEwen BS (2005). Stressed or stressed out: what is the difference?. J Psychiatry Neurosci.

[14] Briere J, Hodges M, Godbout N (2010). Traumatic stress, affect dysregulation, and dysfunctional avoidance: a structural equation model. J Trauma Stress.

[15] Heim C, Ehlert U, Hellhammer DH (2000). The potential role of hypocortisolism in the pathophysiology of stress-related bodily disorders. Psychoneuroendocrinology..

[16] Van der Kolk BA, Pelcovitz D, Roth S, Mandel FS. Dissociation, somatization, and affect dysregulation: the complexity of adaption to trauma. Am J Psychiatry. 1996. 10.1176/ajp.153.7.83 8659645

[17] Heim C, Nemeroff CB (2001). The role of childhood trauma in the neurobiology of mood and anxiety disorders: preclinical and clinical studies. Biol Psychiatry.

[18] Hunter AL, Minnis H, Wilson P (2011). Altered stress responses in children exposed to early adversity: a systematic review of salivary cortisol studies. Stress..

[19] Gatchel RJ, Peng YB, Peters ML, Fuchs PN, Turk DC (2007). The biopsychosocial approach to chronic pain: scientific advances and future directions. Psychol Bull.

[20] Dominick CH, Blyth FM, Nicholas MK (2012). Unpacking the burden: understanding the relationships between chronic pain and comorbidity in the general population. Pain..

[21] Schore AN (2005). Attachment, affect regulation, and the developing right brain: linking developmental neuroscience to pediatrics. Pediatr Rev.

[22] Van der Kolk BA (2006). Clinical implications of neuroscience research in PTSD. Ann N Y Acad Sci.

[23] Ogden P, Minton K, Pain C, van der Kolk B. Trauma and the body: a sensorimotor approach to psychotherapy (Norton series on interpersonal neurobiology). New York: WW Norton & Company; 2006.

[24] Emerson D, Hopper E, Levine PA, Cope S, Van Der Bessel, Kolk M. Overcoming trauma through yoga: Reclaiming your body: North Atlantic Books; 2011.

[25] Emerson D, Sharma R, Chaudhry S, Turner J (2009). Trauma-sensitive yoga. Principles, practice, and research. Int J Yoga Therap.

[26] Price M, Spinazzola J, Musicaro R, Turner J, Suvak M, Emerson D, van der Kolk B (2017). Effectiveness of an extended yoga treatment for women with chronic posttraumatic stress disorder. J Altern Complement Med.

[27] van der Kolk BA, Stone L, West J, Rhodes A, Emerson D, Suvak M, Spinazzola J (2014). Original research yoga as an adjunctive treatment for posttraumatic stress disorder: a randomized controlled trial. J Clin Psychiatry.

[28] Cramer H, Ward L, Saper R, Fishbein D, Dobos G, Lauche R (2015). The safety of yoga: a systematic review and meta-analysis of randomized controlled trials. Am J Epidemiol.

[29] Posadzki P, Ernst E, Terry R, Lee MS (2011). Is yoga effective for pain? A systematic review of randomized clinical trials. Complement Ther Med.

[30] Cramer H, Lauche R, Haller H, Dobos G (2013). A systematic review and meta-analysis of yoga for low back pain. Clin J Pain.

[31] Yoga for pain care Australia. https://yogaforpaincare.com/tag/pain-sensitive-yoga-classes. Accessed 20 Jan 2019.

[32] Moseley GL, Flor H (2012). Targeting cortical representations in the treatment of chronic pain a review. Neurorehabil Neural Repair.

[33] Vlaeyen J, Morley S, Linton SJ, Boersma K, de Jong J. Pain-related fear: exposure based treatment for chronic pain: IASP Press; 2012.

[34] Matthews KA, Wing RR, Kuller LH, Meilahn EN, Kelsey SF, Costello EJ, Caggiula AW (1990). Influences of natural menopause on psychological characteristics and symptoms of middle-aged healthy women. J Consult Clin Psychol.

[35] Newhart MR (2013). Menopause matters: the implications of menopause research for studies of midlife health. Health Sociol Rev.

[36] Cleeland C (2009). The brief pain inventory user guide.

[37] Mease PJ, Spaeth M, Clauw DJ, Arnold LM, Bradley LA, Russell IJ, Kajdasz DK, Walker DJ, Chappell AS (2011). Estimation of minimum clinically important difference for pain in fibromyalgia. Arthritis Care Res.

[38] Anderson KO, Cohen MZ, Mendoza TR, Guo H, Harle MT, Cleeland CS (2006). Brief cognitive-behavioral audiotape interventions for cancer-related pain. Cancer.

[39] Carson JW, Keefe FJ, Lynch TR, Carson KM, Goli V, Fras AM, Thorp SR (2005). Loving-kindness meditation for chronic low back pain results from a pilot trial. J Holist Nurs.

[40] Hsu MC, Schubiner H, Lumley MA, Stracks JS, Clauw DJ, Williams DA (2010). Sustained pain reduction through affective self-awareness in fibromyalgia: a randomized controlled trial. J Gen Intern Med.

[41] Sculco AD, Paup DC, Fernhall B, Sculco MJ (2001). Effects of aerobic exercise on low back pain patients in treatment. Spine J.

[42] Norris FH, Hamblen JL, Wilson JP, Keane TM, Martin T (2004). Standardized self-report measures of civilian trauma and PTSD. Assessing psychological trauma and PTSD.

[43] Hewett ZL, Cheema BS, Pumpa KL, Smith CA (2015). The effects of Bikram yoga on health: critical review and clinical trial recommendations. Evid Based Complement Alternat Med.

[44] Tracy BL, Hart CE (2013). Bikram yoga training and physical fitness in healthy young adults. J Strength Cond Res.

[45] Thompson W, Gordon N, Pescatello L (2009). American College of Sport Medicine. ACSM’s Guidelines for exercise testing and prescription. 8. painos.

[46] Pate JL, Buono MJ (2014). The physiological responses to bikram yoga in novice and experienced practitioners. Altern Ther Health Med.

[47] Adrenaline HIT™. http://www.adrenalinehit.com.au. Accessed 20 Jan 2019.

[48] Ward L, Stebbings S, Sherman KJ, Cherkin D, Baxter GD (2014). Establishing key components of yoga interventions for musculoskeletal conditions: a Delphi survey. BMC Complement Altern Med.

[49] Medicine ACoS (2006). ACSM’s guidelines for exercise testing and prescription.

[50] Hayden JA, Van Tulder MW, Tomlinson G (2005). Systematic review: strategies for using exercise therapy to improve outcomes in chronic low back pain. Ann Intern Med.

[51] Kirkwood G, Rampes H, Tuffrey V, Richardson J, Pilkington K (2005). Yoga for anxiety: a systematic review of the research evidence. Br J Sports Med.

[52] Lecrubier Y, Sheehan D, Weiller E, Amorim P, Bonora I, Sheehan KH, Janavs J, Dunbar G (1997). The MINI international neuropsychiatric interview (MINI). A short diagnostic structured interview: reliability and validity according to the CIDI. Eur Psychiatry.

[53] Tan G, Jensen MP, Thornby JI, Shanti BF (2004). Validation of the brief Pain inventory for chronic nonmalignant pain. J Pain.

[54] Dworkin RH, Turk DC, Wyrwich KW, Beaton D, Cleeland CS, Farrar JT, Haythornthwaite JA, Jensen MP, Kerns RD, Ader DN (2008). Interpreting the clinical importance of treatment outcomes in chronic pain clinical trials: IMMPACT recommendations. J Pain.

[55] McCallum J (1995). The SF-36 in an Australian sample: validating a new, generic health status measure. Aust J Public Health.

[56] Ware JE (2004). SF-36 health survey update.

[57] Lovibond S, Lovibond PF. Manual for the depression anxiety stress scales: Psychology Foundation of Australia; 1996.

[58] Antony MM, Bieling PJ, Cox BJ, Enns MW, Swinson RP (1998). Psychometric properties of the 42-item and 21-item versions of the depression anxiety stress scales in clinical groups and a community sample. Psychol Assess.

[59] American Psychiatric Association. Diagnostic and statistical manual of mental disorders, text revision (DSM-IV-TR): American Psychiatric Association; 2000.

[60] Luxenberg T, Spinazzola J, Van der Kolk B (2001). Complex trauma and disorders of extreme stress (DESNOS) diagnosis, part one: assessment. Dir Psychiatry.

[61] Chesney MA, Neilands TB, Chambers DB, Taylor JM, Folkman S (2006). A validity and reliability study of the coping self-efficacy scale. Br J Health Psychol.

[62] Ungerer O, Deter H-C, Fikentscher E, Konzag TA (2010). Improved diagnostics of trauma-related disease through the application of the life-stressor checklist. Psychother Psychosom Med Psychol.

[63] Baer RA, Smith GT, Hopkins J, Krietemeyer J, Toney L (2006). Using self-report assessment methods to explore facets of mindfulness. Assessment..

[64] Tracy LM, Ioannou L, Baker KS, Gibson SJ, Georgiou-Karistianis N, Giummarra MJ (2016). Meta-analytic evidence for decreased heart rate variability in chronic pain implicating parasympathetic nervous system dysregulation. Pain..

[65] Leon AC, Davis LL, Kraemer HC (2011). The role and interpretation of pilot studies in clinical research. J Psychiatr Res.

[66] Hopkins LB, Medina JL, Baird SO, Rosenfield D, Powers MB, Smits JA (2016). Heated hatha yoga to target cortisol reactivity to stress and affective eating in women at risk for obesity-related illnesses: a randomized controlled trial. J Consult Clin Psychol.

[67] Wieland LS, Skoetz N, Manheimer E, Pilkington K, Vempati R, Berman BM. Yoga treatment for chronic non-specific low-back pain: The Cochrane Library; 2013. 10.1002/14651858.CD010671.pub2 PMC5294833 28076926

[68] Cousineau D, Chartier S (2010). Outliers detection and treatment: a review. Int J Psychol Res.

[69] Sedgwick P, Greenwood N (2015). Understanding the Hawthorne effect. BMJ..

[70] Geneen LJ, Moore A, Clarke C, Martin D, Colvin LA, Smith BH. Physical activity and exercise for chronic pain in adults: an overview of cochrane reviews. Cochrane Database Syst Rev. 2017. 10.1002/14651858.CD011279.pub2 PMC6469540 28087891

[71] Salmon P (2001). Effects of physical exercise on anxiety, depression, and sensitivity to stress: a unifying theory. Clin Psychol Rev.

[72] Ware JE, Gandek B (1998). Overview of the SF-36 health survey and the international quality of life assessment (IQOLA) project. J Clin Epidemiol.

[73] Weber CS, Thayer JF, Rudat M, Wirtz PH, Zimmermann-Viehoff F, Thomas A, Perschel FH, Arck PC, Deter HC (2010). Low vagal tone is associated with impaired post stress recovery of cardiovascular, endocrine, and immune markers. Eur J Appl Physiol.

[74] Kim H-G, Cheon E-J, Bai D-S, Lee YH, Koo B-H (2018). Stress and heart rate variability: a meta-analysis and review of the literature. Psychiatry Investig.

[75] Wulsin LR, Horn PS, Perry JL, Massaro JM, D'agostino RB (2015). Autonomic imbalance as a predictor of metabolic risks, cardiovascular disease, diabetes, and mortality. J Clin Endocrinol Metab.

[76] Zhou X, Ma Z, Zhang L, Zhou S, Wang J, Wang B, Fu W (2016). Heart rate variability in the prediction of survival in patients with cancer: a systematic review and meta-analysis. J Psychosom Res.

[77] Beauchaine TP, Thayer JF (2015). Heart rate variability as a transdiagnostic biomarker of psychopathology. Int J Psychophysiol.

[78] Williams DP, Cash C, Rankin C, Bernardi A, Koenig J, Thayer JF (2015). Resting heart rate variability predicts self-reported difficulties in emotion regulation: a focus on different facets of emotion regulation. Front Psychol.

[79] Kleiger RE, Miller JP, Bigger JT, Moss AJ (1987). Decreased heart rate variability and its association with increased mortality after acute myocardial infarction. Am J Cardiol.

[80] Visnovcova Z, Calkovska A, Tonhajzerova I (2013). Heart rate variability and electrodermal activity as noninvasive indices of sympathovagal balance in response to stress. Acta Med Mart.

[81] Borsook D, Maleki N, Becerra L, McEwen B (2012). Understanding migraine through the lens of maladaptive stress responses: a model disease of allostatic load. Neuron..

[82] Seeman TE, Singer BH, Rowe JW, Horwitz RI, McEwen BS (1997). Price of adaptation—allostatic load and its health consequences: MacArthur studies of successful aging. Arch Intern Med.

[83] McEwen BS (2002). Sex, stress and the hippocampus: allostasis, allostatic load and the aging process. Neurobiol Aging.

[84] Lindheimer JB, O’Connor PJ, Dishman RK (2015). Quantifying the placebo effect in psychological outcomes of exercise training: a meta-analysis of randomized trials. Sports Med.

[85] Papp ME, Lindfors P, Storck N, Wändell PE (2013). Increased heart rate variability but no effect on blood pressure from 8 weeks of hatha yoga–a pilot study. BMC Res Notes.

[86] Parsons HM (1974). What happened at Hawthorne?: new evidence suggests the Hawthorne effect resulted from operant reinforcement contingencies. Science..

[87] Moseley L (2002). Combined physiotherapy and education is efficacious for chronic low back pain. Aust J Physiother.

[88] Du S, Yuan C, Xiao X, Chu J, Qiu Y, Qian H (2011). Self-management programs for chronic musculoskeletal pain conditions: a systematic review and meta-analysis. Patient Educ Couns.

[89] Mist SD, Firestone KA, Jones KD (2013). Complementary and alternative exercise for fibromyalgia: a meta-analysis. J Pain Res.

[90] Nijs J, Kosek E, Van Oosterwijck J, Meeus M (2012). Dysfunctional endogenous analgesia during exercise in patients with chronic pain: to exercise or not to exercise?. Pain Physician.

[91] Chang DG, Holt JA, Sklar M, Groessl EJ (2016). Yoga as a treatment for chronic low back pain: a systematic review of the literature. J Orthop Rheumatol.

[92] Holtzman S, Beggs RT (2013). Yoga for chronic low back pain: a meta-analysis of randomized controlled trials. Pain Res Manag.

[93] Ward L, Stebbings S, Cherkin D, Baxter GD (2013). Yoga for functional ability, Pain and psychosocial outcomes in musculoskeletal conditions: a systematic review and meta-analysis. Musculoskeletal Care.

[94] Saper RB, Boah AR, Keosaian J, Cerrada C, Weinberg J, Sherman KJ (2013). Comparing once-versus twice-weekly yoga classes for chronic low back pain in predominantly low income minorities: a randomized dosing trial. Evid Based Complement Alternat Med.

[95] Sherman KJ, Cherkin DC, Wellman RD, Cook AJ, Hawkes RJ, Delaney K, Deyo RA (2011). A randomized trial comparing yoga, stretching, and a self-care book for chronic low back pain. Arch Intern Med.

[96] Tilbrook HE, Cox H, Hewitt CE, Kang'ombe AR, Chuang L-H, Jayakody S, Aplin JD, Semlyen A, Trewhela A, Watt I (2011). Yoga for chronic low back pain: a randomized trial. Ann Intern Med.

[97] Williams KA, Petronis J, Smith D, Goodrich D, Wu J, Ravi N, Doyle EJ, Juckett RG, Kolar MM, Gross R (2005). Effect of Iyengar yoga therapy for chronic low back pain. Pain.

