# Supplementary material for: #MindinBody - feasibility of vigorous exercise (Bikram yoga versus high intensity interval training) to improve persistent pain in women with a history of trauma: a pilot randomized control trial
Source: BMC Complement Altern Med. 2019 Aug 29;19:234. doi: 10.1186/s12906-019-2642-1 (PMC6714085; doi:10.1186/s12906-019-2642-1)
Supplement: Supplementary file 1 — Table S1. Total sample (N = 32) baseline means (M) and standard deviations (SD) of primary and secondary outcome measures and correlations with persistent pain levels (Brief Pain Inventory Total (BPI TOT]) (DOCX 19 kb) [file 12906_2019_2642_MOESM1_ESM.docx]

Additional file 1: Table S1 Total sample (N= 32) baseline means (M) and standard deviations (SD) of primary and secondary outcome measures and correlations with persistent pain levels (Brief Pain Inventory Total (BPI TOT])

|  | M | SD | Range | r with BPI TOT | P-value |
| --- | --- | --- | --- | --- | --- |
| Primary Outcome |  |  |  |  |  |
| BPI TOTAL | 7.94 | 3.63 | 1.43 - 17.29 | na | na |
| BPI-Severity | 3.88 | 1.60 | 0.75 - 8.00 | na | na |
| BPI-Interference | 3.96 | 2.17 | 0.43 - 9.29 | na | na |
| Secondary Outcomes |  |  |  |  |  |
| SF-36 PF | 73.59 | 18.28 | 40.00 – 100 | -0.633 | p < 0.001 |
| SF-36 RP | 54.30 | 24.51 | 18.75 - 100 | -0.531 | 0.002 |
| SF-36 BP | 44.41 | 17.81 | 21.00 - 74.00 | -0.588 | p < 0.001 |
| SF-36 GH | 30.43 | 21.13 | 0.00 - 68.75 | -0.535 | 0.002 |
| SF-36 VT | 29.69 | 20.82 | 0.00 - 68.75 | -0. 430 | 0.014 |
| SF-36 SF | 51.17 | 24.46 | 0.00 - 100.00 | -0.394 | 0.026 |
| SF-36 RE | 51.04 | 27.82 | 8.33 - 91.67 | -0.298 | 0.098 |
| SF-36 MH | 50.78 | 20.20 | 0.00 - 80.00 | -0.404 | 0.022 |
| DASS-21 Stress | 11.09 | 4.25 | 3.00 - 8.00 | 0.264 | 0.144 |
| DASS-21 Anxiety | 6.66 | 4.74 | 0.00 - 17.00 | 0.330 | 0.065 |
| DASS-21 Depression | 9.00 | 5.32 | 0.00 - 21.00 | 0.297 | 0.099 |
| LSC-R number events | 5.13 | 3.92 | 1 - 14 | 0.377 | 0.033 |
| SIDES-SR total | 14.03 | 11.27 | 0.00 - 39.42 | 0.179 | 0.327 |
| FFMQ total | 116.31 | 20.12 | 38 – 226 | -0.104 | 0.571 |
| CSE | 117.41 | 47.78 | 83 – 152 | -0.261 | 0.150 |
| HR | 69 | 12 | 50 - 98 | 0.417 | 0.017 |
| SDNN | 59.57 | 27.42 | 24.89 - 132.30 | -0.076 | 0.679 |
| LFnu | 49.35 | 16.90 | 24.90 - 93.98 | -0.118 | 0.519 |
| HFnu | 47.60 | 15.91 | 6.09 – 74.72 | 0.037 | 0.841 |
| SAP | 110 | 9 | 94 - 129 | 0.057 | 0.755 |
| DAP | 72 | 11 | 53 - 100 | 0.029 | 0.873 |
| Baseline Spearman’s correlations with BPI TOTAL score are reported for the primary and all secondary outcome measures  Abbreviations: SD: standard deviation; BPI: Brief Pain Inventory; PF: Physical Functioning; RP: Role Physical; BP: Body Pain; GH: General Health; VT: Vitality; SF: Social Functioning; RE: Role Emotional; MH: Mental Health; DASS: Depression, Anxiety, and Stress Scale; SIDES-SR: Self-Report Instrument for Disorders of Extreme Stress; FFMQ: Five Factor Mindfulness Scale; CSE: Coping Self-Efficacy Scale; HR: Heart Rate; HRV: Heart Rate Variability; SDNN: Standard Deviation of the Normal beat to Normal beat interval; LFnu: Low-Frequency normalized units; HFnu: High-Frequency normalized units; SAP: systolic arterial pressure; DAP: diastolic arterial pressure | | | | | |
